# Promising Loci and Genes for Yolk and Ovary Weight in Chickens Revealed by a Genome-Wide Association Study

**DOI:** 10.1371/journal.pone.0137145

**Published:** 2015-09-02

**Authors:** Congjiao Sun, Jian Lu, Guoqiang Yi, Jingwei Yuan, Zhongyi Duan, Lujiang Qu, Guiyun Xu, Kehua Wang, Ning Yang

**Affiliations:** 1 National Engineering Laboratory for Animal Breeding and MOA Key Laboratory of Animal Genetics and Breeding, College of Animal Science and Technology, China Agricultural University, Beijing, China; 2 Jiangsu Institute of Poultry Science, Yangzhou, Jiangsu, China; Institut Jacques Monod, FRANCE

## Abstract

Because it serves as the cytoplasm of the oocyte and provides a large amount of reserves, the egg yolk has biological significance for developing embryos. The ovary and its hierarchy of follicles are the main reproductive organs responsible for yolk deposition in chickens. However, the genetic architecture underlying the yolk and ovarian follicle weights remains elusive. Here, we measured the yolk weight (YW) at 11 age points from onset of egg laying to 72 weeks of age and measured the follicle weight (FW) and ovary weight (OW) at 73 weeks as part of a comprehensive genome-wide association study (GWAS) in 1,534 F_2_ hens derived from reciprocal crosses between White Leghorn (WL) and Dongxiang chickens (DX). For all ages, YWs exhibited moderate single nucleotide polymorphism (SNP)-based heritability estimates (0.25–0.38), while the estimates for FW (0.16) and OW (0.20) were relatively low. Independent univariate genome-wide screens for each trait identified 12, 3, and 31 novel significant associations with YW, FW, and OW, respectively. A list of candidate genes such as *ZAR1*, *STARD13*, *ACER1b*, *ACSBG2*, and *DHRS12* were identified for having a plausible function in yolk and follicle development. These genes are important to the initiation of embryogenesis, lipid transport, lipoprotein synthesis, lipid droplet promotion, and steroid hormone metabolism, respectively. Our study provides for the first time a genome-wide association (GWA) analysis for follicle and ovary weight. Identification of the promising loci as well as potential candidate genes will greatly advance our understanding of the genetic basis underlying dynamic yolk weight and ovarian follicle development and has practical significance in breeding programs for the alteration of yolk weight at different age points.

## Introduction

Chicken egg yolk is an emulsion of water (48%), lipids (33%), and proteins (17%) [1]. Because it serves as the cytoplasm of the oocyte and provides a large amount of reserves, egg yolk functions biologically to provide the above-mentioned nutrients to the developing embryos [2],and yolk can accumulate significant amounts of IgY immunoglobulins (up to 100 mg per egg) to provide innate immunity to the embryos [3]. Egg yolk is widely used in the food industry for its high nutritional value to humans [4]. Furthermore, the bioactive substances of egg yolk are applied in the pharmaceutical and cosmetics fields for their binding properties, emulsion stability, and natural antioxidants [5–7].

The central area of the chicken ovary is composed of a vascularized medulla and a cortex containing the small follicles that are oocytes covered by follicular epithelium [2], and egg yolk is formed in these ovarian follicles by the consecutive deposition of lipids and proteins [8]. The sequential development of oocytes in ovaries leads to the display of a hierarchy in the follicles with four to six yolky follicles of gradually increasing size at the surface. Yolk precursors, however, are not synthesized in the ovary but are produced by the liver and then transported in the blood to the ovarian oocytes [2, 9]. Vitellogenin, consisting of one phosvitin and two lipovitellins, is the main carrier for protein transportation from the liver to the ovary in the blood [10]. The lipid carrier is very low-density lipoprotein (VLDL), which has a standard structure consisting of a core of triglycerides and cholesterol esters surrounded by a surface layer composed of phospholipids, cholesterol, and apoproteins [11]. Yolk precursors (vitellogenin and VLDL) are transported in the follicular wall and are released near the basolateral membrane of the follicles. Then the penetration of these precursors is ensured through a process of endocytosis induced by the receptor LR8 for the deposition of yolk [12, 13].

Due to the wide utilization of egg yolk, many efforts have been performed to alter egg yolk weight [14]. However, egg yolk weight is a complicated quantitative trait affected by many factors, such as breed and hen age [14]. The yolk weight is increased with the age of the laying birds; for eggs of the same size, old hens produce larger egg yolks than young hens, and the albumen weight is correspondingly decreased [15]. The strategy of identifying the quantitative trait loci (QTLs) or causal genes that are related to yolk formation and ovarian follicle development is a powerful tool to illustrate the genetic control for yolk weight and follicle development. A decade ago, microsatellite markers were employed to detect the causal regions associated with yolk weight, and multiple QTLs were reported [16–19]. Until now, however, only seven QTLs(distributed on chromosomes 4, 6, 9, 11, 15, 22, and 26) that relate to yolk weight had been identified. And these QTLs, which have poor repeatability and wide positional confidence intervals, were deposited in the AnimalQTL Database (http://www.animalgenome.org/cgi-bin/QTLdb/index). In recent years, genome-wide association studies (GWASs) have been utilized to identify the associations between genomic loci and phenotypes with relatively high-density single nucleotide polymorphism (SNP) arrays in chickens [17]. Recently, with the rapid advance in next-generation sequencing technologies, large numbers of SNPs have been discovered in chickens [20]. The development of the Affymetrix 600K Chicken SNP array allows further efficient screening for causal loci and genes with relevance to target traits.

In the current study, we conducted GWA analysis on the yolk weight at 11 time points from the onset of egg laying to 72 weeks utilizing 600K high-density SNP arrays in an F_2_ chicken population to explore the associated genomic loci and genes that contribute to the dynamic change in the yolk weight with the aging process. Furthermore, the GWA analysis was also performed on ovary and follicle weights at 73weeksof age to detect the causal genes related to follicle and ovary development. This study lays the foundation for an investigation into the genetic control of yolk and follicle development.

## Materials and Methods

### Ethics statement

All protocols and procedures involving animals were performed in accordance with the Guidelines for the Care and Use of Experimental Animals established by the Ministry of Agriculture of China (Beijing, China). All animal work was approved by the Animal Welfare Committee of China Agricultural University (permit number: SYXK 2007–0023).

### Resource population

Reciprocal crosses between standard breed White Leghorns (WL) and Dongxiang chickens (DX), an indigenous Chinese strain, were utilized to develop an F_2_ resource population. For parents, six WL and six DX males were initially mated with 133 DX and 80 WL females, generating 1,029 and 552 chicks for the F_1_ generation, respectively. Then 25 males and 407 females from the WL/DX cross and 24 males and 235 females from the DX/WL cross in the F_1_ generation were used to produce the F_2_ population, which consisted of 3,749 chicks in a single hatch, originating from 49 half-sib and 590 full-sib families. The hens were kept in individual cages of the same environment with food and water *adlibitum* at the Jiangsu Institute of Poultry Sciences. Finally, 1,534 hens from 49 half-sib families and 550 full-sib families with sufficient phenotypic and pedigree information were selected for SNP genotyping. All layers were caged individually and subjected to a light/dark cycle of 16h of light and 8h of darkness (16L:8D)and raised in the same environment with feed and water *adlibitum*.

### Phenotypic measurements

Yolk weight (YW) was measured for the first egg of each hen and again at 32, 36, 40, 44, 48, 52, 56, 60, 66, and 72 weeks of age, consisting of 11 age points. Except for the first egg, fresh eggs were collected for four successive days to ensure two eggs per hen at each age point. Eggs were broken to obtain the internal contents, and then the yolk was separated from the albumen and weighed by electronic scale. All hens were humanely sacrificed by 60%–70% carbon dioxide at 73weeks of age, the follicles and ovaries were then separated and weighted for follicle-active hens. FW includes the weight of all follicles that were contained in the ovary, and OW indicates the weight of the follicle-free ovary, which consisted of a highly vascularized central area (medulla) and a peripheral area (cortex). Descriptive statistics were calculated with R project (R version 3.1.2) using all available records. The function of ‘rntransform’ in the GenABEL package of R project was used for the rank-based inverse normal transformations [21].

### Genotyping and quality control

Genomic DNA was extracted by the standard phenol/chloroform method and genotyped with the Affymetrix 600K Axiom Chicken Genotyping Array (Affymetrix, Inc. Santa Clara, CA, USA). Affymetrix Power Tools v1.16.0 (APT) software was then used for quality control (QC) and genotype calling. Specifically, only samples with dish quality control (DQC) >0.82 and a call rate>97% were included in the subsequent analyses. An R script supplied by Affymetrix was run to compute the SNP QC metrics and filter out individual SNPs falling below given thresholds. After these QC steps, 1,512 individuals and 532,299 SNPs remained valid. Because the current statistical methods are more powerful for the detection of the associations between phenotypes and autosomal genotypes, all SNPs on sex chromosomes were excluded in the QC process. The PLINK v1.90 package [22] was then used for further quality control to improve the detection power, in which SNPs with a minor allele frequency (MAF)<5% and Hardy—Weinberg equilibrium (HWE) test *P*< 1 × 10^−6^ were removed from the downstream analysis. Some sporadic missing genotypes were imputed using the BEAGLE v4.0 procedure [23], and only SNPs with an imputation quality score *R* ^2^>0.5 were retained. Ultimately, up to 1,512 individuals and 435,867 SNPs were valid for the GWA analysis.

### Estimation of heritability and contribution to phenotypic variance

A univariate restricted maximum likelihood (REML) estimation was implemented in the GCTA v1.24 program [24] to estimate the heritability explained by the eligible SNPs (*h* ^*2*^_*snp*_). We also quantified the pair-wise genetic and phenotypic correlations for each trait at multiple time points with a bivariate mixed model [25]. A genetic relationship matrix (GRM) derived from all genotyped SNPs on autosomes and two linkage groups was created, and the top three PCs calculated by the GCTA tool were included as covariates to account for the potential population structure. We then estimated the contribution to phenotypic variance (CPV)made by these associated loci or genomic regions.

### Genome-wide association analysis

The principal component analysis (PCA) implemented in the PLINK package was conducted prior to genome-wide association (GWA) analysis to eliminate spurious associations resulting from the presence of cryptic relatedness or hidden population stratification. To properly decide the thresholds for genome-wide significant/suggestive associations, we used the simpleM method [26] to correct the number of multiple tests. With this approach, an effective number of 59,308 independent tests was suggested; hence, the genome-wide significant and suggestive *P* values were 8.43 × 10^−7^ (0.05/59,308) and 1.69 × 10^−5^ (1.00/59,308), respectively.

A univariate association test equipped with an exact mixed model approach in GEMMA v0.94 software [27] was performed with the valid individuals and SNPs. A centered relatedness matrix was calculated with the independent SNPs, and then the derived Wald test *P* value was calculated for the significance level between the SNPs and phenotypes. The univariate linear mixed model as follows:

y = Wα+xβ+u+ε

For the equation, **y** is an *n* × 1 vector of phenotypic values for *n* individuals, **W** is an *n* × *5* matrix of covariates (fixed effects, *i*.*e*., top three PCs, sire and dam effects) including a column vector of 1, **α** is a *5* × 1 vector of corresponding coefficients including the intercept, **x** is an *n* × 1 vector of marker genotypes at the locus tested, *β* is the corresponding effect size of the marker and all effects are reported for the minor allele in each marker, **u** is an *n* × 1 vector of random polygenic effects with a covariance structure as **u**~ N(0, **K**V_*g*_), where **K** represents a known *n* × *n*genetic relatedness matrix derived from SNP markers and V_*g*_ is the polygenic additive variance, and **ε** is an *n* × 1 vector of random residuals with **ε** ~ N(0, **I**V_*e*_), where **I** is an *n* × *n* identity matrix, and V_*e*_ is the residual variance component. We used the Wald test statistic $F_{Wald}\;=\;{\hat{\beta }}^{2}/Var(\hat{\beta })$ for each SNP to test the null hypothesis *β* = 0, where the best linear unbiased estimate (BLUE) of *β* and the corresponding sampling variance $Var(\hat{\beta })$ are obtained by solving the mixed model equations (MME) based on estimated V_*g*_ and V_*e*_.

The Manhattan plots and quantile—quantile (QQ) plots were drawn by the “gap” packages [28] in R project. The genomic inflation factor λ, which is the judgment of the extent of false positive signals, was also calculated with the function of estlambda implemented in the GenABEL package [21] in R project.

### Linkage disequilibrium analysis

Notably, many SNPs maybe passively significantly associated with target traits, resulting from their linkage to a strongly causal mutant. In general, GWAS does not distinguish a genuine causal locus from those that are statistically significant loci within a strong linkage disequilibrium (LD) region. Therefore, in order to characterize the potential candidate genes responsible for a trait, we conducted an LD analysis and inferred the haplotype blocks containing peak SNPs by Haploview v4.2 [29]. A block is derived using the solid spine algorithm, and defined as that the first and last SNPs in a region that is strong in LDs (D′ ≥ 0.8) with all intermediate SNPs.

### Gene identification

Variant Effect Predictor (VEP) and BioMart tools based on the galGal4 assembly supported by Ensembl were used for the identification of candidate genes in which significant loci were located [30] and for the detection of the genes in a given genomic region [31].

## Results

### Phenotype description and genetic parameters

Descriptive statistics for egg yolk weights at 11 age points and follicle and ovary weights at 73 weeks were presented in Table 1. We observed a curvilinear increase in yolk weights with advancing hen age from the onset of egg laying to 66 weeks and a slight decrease at 72 weeks. Follicle and ovary weights were measured for 1,429 follicle-active hens (1,508 slaughtered in total) at 73 weeks old. These two traits exhibited large coefficients of variation, which may be due to the different oviposition intervals for old hens. All phenotypic values conformed to the normal distribution after rank-based inverse normal transformation.

**Table 1:** Descriptive statistics of yolk and ovary traits for the F_2_ population.

Trait(weight)	N	Mean (g)	SD	CV(%)	Max	Min
**FYW**	1,494	9.04	0.96	10.58	13.00	6.10
**YW32**	1,473	13.75	1.04	7.57	17.10	9.40
**YW36**	1,464	14.54	1.21	8.30	18.70	10.50
**YW40**	1,476	15.31	1.23	8.04	19.50	11.50
**YW44**	1,420	15.82	1.40	8.86	20.70	10.50
**YW48**	1,226	16.01	1.37	8.58	20.80	12.00
**YW52**	1,225	16.42	1.43	8.72	21.80	11.70
**YW56**	1,348	16.65	1.58	9.52	22.90	11.30
**YW60**	1,364	16.96	1.55	9.15	22.90	12.90
**YW66**	1,304	16.97	1.67	9.87	24.90	11.20
**YW72**	1,253	15.62	1.73	11.08	21.80	7.30
**FW**	1,439	29.72	10.37	35.17	60.30	2.12
**OW**	1,439	4.23	1.50	35.72	14.31	1.53

Abbreviations: FW = follicle weight; FYW = yolk weight of first egg; N = number of samples; OW = ovary weight; SD = standard deviation; YW = yolk weight; YW32, YW36, YW40, YW44, YW48, YW52, YW56, YW60, YW66, and YW72 = yolk weight of 32, 36, 40, 44, 48, 52, 56, 60, 66, and 72 weeks of age, respectively.

We quantified the additive genetic variation in liability to yolk weights at the different ages captured by eligible GWAS markers. Univariate GCTA analyses revealed that all yolk weights had moderate heritable patterns (Table 2), and the highest SNP-based heritability estimate was found in YW40 (*h* ^*2*^ = 0.38). Moreover, bivariate GCTA analyses indicated that yolk weights at the various ages exhibited highly and positively correlation, especially for yolk weights at neighboring time points. At the beginning of the entire laying stage, the yolk weight of the first egg (FYW) showed slightly lower genetic correlations with the yolk weights at the following ages (*r*_*g*_<0.60), compared with those among yolk weights from 32 to 72 weeks of age (*r*_*g*_>0.80). Notably, the follicle weight at 73 weeks had low phenotypic (0.02 to 0.17) and genetic (-0.003 to 0.52) correlation with yolk weights at the 11 age points. Similarly, the ovary weight also poorly correlated with yolk weights, and the phenotypic and genetic correlation coefficients ranged from 0.02 to 0.13 and -0.02 to 0.40, respectively. However, the genetic correlation between ovary weight and follicle weight was relatively high (0.86).

**Table 2:** Genetic parameters of yolk and ovary weights.

Traits	FYW	YW32	YW36	YW40	YW44	YW48	YW52	YW56	YW60	YW66	YW72	FW	OW
**F YW**	**0.34(0.04)**	0.52(0.09)	0.58(0.09)	0.32(0.10)	0.35(0.11)	0.52(0.11)	0.46(0.11)	0.35(0.12)	0.44(0.10)	0.29(0.12)	0.29(0.11)	-0.003(0.14)	-0.02(0.13)
**YW32**	0.28	**0.34(0.04)**	1.00(0.02)	0.99(0.02)	0.97(0.03)	0.99(0.04)	0.82(0.07)	0.81(0.08)	0.80(0.06)	0.80(0.07)	0.80(0.07)	0.52(0.12)	0.35(0.12)
**YW36**	0.24	0.61	**0.31(0.04)**	1.00(0.02)	1.00(0.03)	0.99(0.04)	0.88(0.06)	0.87(0.07)	0.92(0.05)	0.86(0.06)	0.85(0.06)	0.34(0.14)	0.40(0.12)
**YW40**	0.22	0.64	0.63	**0.38(0.04)**	0.99(0.02)	1.00(0.03)	0.91(0.04)	0.93(0.05)	0.96(0.04)	0.83(0.05)	0.87(0.05)	0.34(0.16)	0.34(0.12)
**YW44**	0.18	0.56	0.56	0.65	**0.31(0.04)**	0.99(0.04)	0.92(0.05)	0.95(0.05)	0.98(0.04)	0.89(0.05)	0.86(0.05)	0.45(0.13)	0.35(0.12)
**YW48**	0.21	0.56	0.54	0.63	0.58	**0.27(0.05)**	0.89(0.05)	0.95(0.05)	1.00(0.04)	0.89(0.05)	0.83(0.06)	0.39(0.14)	0.28(0.14)
**YW52**	0.20	0.50	0.50	0.60	0.55	0.61	**0.29(0.05)**	1.00(0.04)	0.97(0.04)	0.90(0.05)	0.91(0.05)	0.22(0.15)	0.21(0.14)
**YW56**	0.18	0.47	0.45	0.57	0.53	0.55	0.61	**0.25(0.04)**	1.00(0.03)	0.91(0.05)	0.97(0.04)	0.32(0.16)	0.35(0.14)
**YW60**	0.19	0.49	0.48	0.59	0.53	0.60	0.60	0.63	**0.31(0.05)**	0.94(0.04)	0.95(0.04)	0.39(0.13)	0.27(0.13)
**YW66**	0.12	0.44	0.46	0.57	0.52	0.56	0.59	0.60	0.60	**0.31(0.05)**	0.96(0.03)	0.15(0.15)	0.17(0.14)
**YW72**	0.12	0.41	0.38	0.50	0.46	0.50	0.50	0.55	0.54	0.58	**0.35(0.05)**	0.19(0.14)	0.19(0.13)
**FW**	0.02	0.14	0.17	0.16	0.13	0.14	0.11	0.07	0.14	0.06	0.10	**0.16(0.04)**	0.86(0.09)
**OW**	0.02	0.12	0.09	0.06	0.08	0.04	0.08	0.07	0.05	0.03	0.05	0.13	**0.20(0.04)**

Diagonal: Heritability estimates. Lower triangle: Phenotypic correlations. Upper triangle: Genetic correlations. Standard errors of the estimates are in parentheses. Abbreviations: FYW = yolk weight of first egg; OW = ovary weight;YW32, YW36, YW40, YW44, YW48, YW52, YW56, YW60, YW66, and YW72 = yolk weight of 32,36,40,44,48,52,56,60,66, and 72 weeks of age, respectively.

### Identification of candidate loci by a genome-wide association study

**Fig 1:**
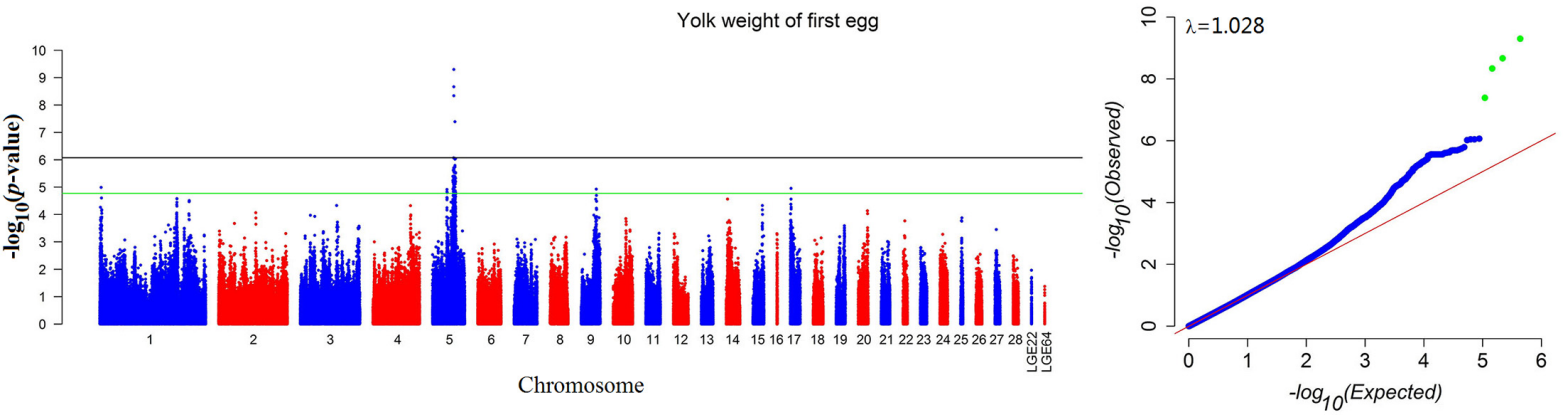
Manhattan plot (left) and quantile-quantile plot (right) of the observed *P* values for the yolk weight of first eggs (FYW). The Manhattan plot indicates the-log_10_ (observed *P* values) for genome-wide SNPs (y-axis) plotted against their respective positions on each chromosome (x-axis), and the horizontal black and green lines depict the genome-wide significant (8.43 × 10^−7^) and suggestive significant (1.69 × 10^−5^) thresholds, respectively. For the quantile-quantile plot, the x-axis shows the expected-log_10_-transformed *P* values, and the y-axis represents the observed-log_10_-transformed *P* values. The genomic inflation factors (λ) are shown on the top left in the QQ plots. Green points represent the genome-wide significant associations.

We conducted separate association tests using a univariate model for egg weights at each age point and follicles and ovary weight at 73weeks. The Manhattan plots and the Quantile-Quantile (Q-Q) plots of the yolk weight of first eggs (FYW) are shown as an example in Fig 1, and the other traits are shown in S1–S12 Figs. The detailed messages for genome-wide significant SNPs are listed in Tables 3 and 4, and the descriptions for suggestive significant SNPs are shown in S1 Table. The CPVs of the loci were estimated by a tool of GCTA and ranged from 2.40 to 3.47% for yolk weights (Table 3) and from 2.11 to 3.76% for ovary weights (Table 4).

**Table 3:** Genome-wide association analyses for yolk weight (YW) and follicle weight (FW).

Traits	SNP ID	Chr	Position	Candidate genes /Nearest Gene	Location	Minor/Majorallele	MAF	N	Effect size^a^ (s.e.m.)	CPV(%)	P value
**FYW**	rs313187645	5	40106943	DOT1L	intron	A/G	0.307	1,494	0.293(0.047)	3.47	5.00E-10
**FYW**	rs312299419	5	40167320	STON2	intron	T/G	0.277	1,494	-0.294(0.049)	3.34	2.16E-09
**FYW**	rs314422825	5	40194495	STON2	exon(missense)	A/G	0.333	1,494	-0.269(0.046)	3.18	4.61E-09
**FYW**	rs315568325	5	42130216	GALC	downstream_1,172bp	T/C	0.288	1,494	-0.266(0.048)	2.80	4.08E-08
**YW40**	rs16187384	23	1456741	RPA2	Intron	A/G	0.493	1,476	-0.241(0.044)	2.77	3.76E-08
**YW48**	rs313116098	1	173851584	BRCA2/ZAR1	exon/downstream_2,140bp	A/G	0.298	1,226	0.242(0.049)	2.48	8.61E-07
**YW56**	rs316715137	3	22975753	EML4/KCNG3	intron/downstream_267kb	A/G	0.291	1,348	-0.263(0.049)	2.61	7.79E-08
**YW56**	rs314923267	3	22971805	EML4/ KCNG3	intron/downstream_267kb	T/C	0.277	1,348	0.256(0.051)	2.40	6.57E-07
**YW66**	rs312474469	28	3667179	ELL	intron	T/C	0.177	1,304	0.289(0.057)	2.49	4.91E-07
**YW66**	rs315213484	28	3665094	ELL	upstream_1,134bp	A/C	0.178	1,304	0.286(0.057)	2.47	6.95E-07
**YW72**	rs312274422	3	79252286	ENSGALG00000028314	downstream_80,003bp	T/G	0.280	1,253	-0.278(0.050)	2.79	3.53E-08
**YW72**	rs312873273	17	1366852	RGS3	intron	C/G	0.213	1,253	-0.298(0.058)	2.89	3.73E-07
**FW**	rs14920313	1	172060693	DCLK1/SPG20	intron/upstream_7.3kb	T/C	0.249	1,450	-0.261(0.051)	2.60	1.09E-07
**FW**	rs14920355	1	172084219	DCLK1	intron	T/C	0.246	1,450	-0.257(0.051)	2.42	3.00E-08
**FW**	rs313140585	1	172453837	NBEA/STARD13	intron/upstream_47.7kb	T/C	0.277	1,450	-0.242(0.048)	2.50	3.88E-07

Abbreviations: Chr = chromosome; CPV = contribution to phenotype variance; FW = follicle weight; FYW = first yolk weight; MAF = minor allele frequency; N = number of samples; SNP = single nucleotide polymorphism; YW40,48,56,66, and 72 = yolk weights of 40,48,56,66,and 72 weeks, respectively.

^a^ Effect size means the effect of minor alleles. Positive/negative effect size value means that the substitution of major allele to minor allele can lead to heavier/lighter yolk or ovary weight.

**Table 4:** Significant single nucleotide polymorphism (SNP) markers associated with ovary weight (OW).

SNP	Position	Candidate genes /Nearest Gene	Location	Minor/Majorallele	MAF	N	Effect size^a^ (s.e.m.)	CPV(%)	*P* value
**Chr 1**									
rs317441976	169051794	DLEU7	upstream(71.3kb)	T/G	0.412	1,450	-0.278(0.048)	3.76	9.65E-09
rs316815773	169670916	NEK5	Intron	T/C	0.489	1,450	0.247(0.046)	3.10	9.49E-08
rs314051078	169046176	DLEU7	upstream(76.9kb)	A/G	0.387	1,450	-0.255(0.048)	3.24	1.16E-07
rs317796161	169018288	DLEU7	upstream(104.8kb)	C/G	0.486	1,450	0.249(0.047)	2.79	1.83E-07
rs15500685	169696167	NEK3	intron	T/C	0.477	1,450	0.240(0.046)	2.96	2.18E-07
rs312737959	169709920	CKAP2	intron	A/G	0.443	1,450	-0.250(0.048)	2.98	2.21E-07
rs13972990	169562091	DHRS12	downstream(6,152bp)	T/C	0.457	1,450	-0.246(0.048)	2.93	3.96E-07
**Chr 28**									
rs315788897	1699155	ACER1b/ACSBG2	intron/intron	T/C	0.144	1,450	0.375(0.059)	3.18	1.70E-10
rs316105069	2123244	SCAMP4	downstream(493bp)	A/G	0.136	1,450	0.382(0.060)	3.14	1.94E-10
rs13663720	1788269	ADAMTS10	intron	T/C	0.155	1,450	0.358(0.059)	3.11	1.89E-09
rs317889060	2012197	DOT1L	intron	T/C	0.228	1,450	0.299(0.051)	2.88	7.43E-09
rs318099911	2163700	ABHD17A	upstream(3,437bp)	T/C	0.143	1,450	0.341(0.059)	2.51	8.30E-09
rs316358363	1626264	ACSBG2	upstream(4,223bp)	A/G	0.161	1,450	0.320(0.056)	2.43	1.11E-08
rs314735191	1514399	NRTN	upstream(4,727bp)	T/C	0.186	1,450	0.298(0.053)	2.57	1.77E-08
rs314625273	2230957	ONECUT3	downstream(2,970bp)	C/G	0.128	1,450	0.350(0.062)	2.44	2.30E-08
rs316444293	2342019	TCF3	intron	A/C	0.159	1,450	0.310(0.057)	2.39	6.18E-08
rs313040427	1712749	ANP32B	intron	T/C	0.165	1,450	0.306(0.056)	2.47	6.73E-08
rs314385559	2367109	TCF3	downstream(1,276bp)	A/G	0.163	1,450	0.302(0.057)	2.3	1.11E-07
rs14305841	2185417	REXO1	intron	T/C	0.133	1,450	0.325(0.061)	2.11	1.19E-07
rs318236639	1929393	OAZ	downstream(777bp)	A/G	0.243	1,450	0.259(0.049)	2.45	1.27E-07
rs16211139	1602011	RFX2	downstream(10.9kb)	C/G	0.239	1,450	0.262(0.049)	2.56	1.33E-07
rs317858034	2042162	AP3D1	intron	T/C	0.212	1,450	0.277(0.053)	2.51	2.10E-07
rs317537249	1843976	ACTL9	downstream(2,988bp)	T/C	0.275	1,450	0.239(0.046)	2.2	2.18E-07
rs313479236	1821752	ZAP70	downstream(4,220bp)	T/C	0.226	1,450	0.263(0.051)	2.47	2.28E-07
rs316676992	1849612	ACTL9/MUC16	upstream(1,333 bp)/downstream(2,188 bp)	A/G	0.275	1,450	0.236(0.046)	2.13	3.30E-07
rs317641108	1926277	OAZ/C19orf35	downstream(3,893 bp)/intron	A/G	0.238	1,450	0.253(0.049)	2.32	3.51E-07
rs315715629	1761595	ADAMTS10	intron	T/C	0.243	1,450	0.246(0.049)	2.21	6.13E-07
rs313972624	1732609	MYO1F	intron	A/C	0.241	1,450	0.245(0.049)	2.21	6.90E-07
rs312587681	2335214	TCF3	intron	T/C	0.158	1,450	0.288(0.058)	2.03	6.99E-07
rs314330209	1990610	DOT1L	intron	A/G	0.274	1,450	0.230(0.046)	2.13	7.26E-07
rs14305824	2179908	REXO1	intron	A/G	0.176	1,450	0.269(0.054)	1.95	7.95E-07

Abbreviations: Chr = chromosome; CPV = contribution to phenotype variance; MAF = minor allele frequency; N = number of samples; SNP = single nucleotide polymorphism.

^a^ Effect size means the effect of minor alleles. Positive/negative effect size value means that the substitution of major allele to minor allele can lead to heavier/lighter yolk or ovary weight.

#### Yolk weight

A total of 11 significant loci distributed on chromosome 1, 3, 5, 17, and 28 were associated with YW at different age points including the age of the hen at the first egg(AFE) and 40, 48, 56, 66,and 72 weeks of age (Table 3). Eleven genes, including *DOT1L*, *STON2*, *GALC*, *RPA2*, *BRCA2*, *ZAR1*, *EML4*, *KCNG3*, *ELL*, *ENSGALG00000028314* and *RGS3F* were detected as harboring or being near these significant loci (Table 3).

#### Follicle weight

Three SNPs located on chromosome 1 (GGA1) were significantly associated with follicle weight (FW). Detailed SNP messages are presented in Table 3. These three significant SNPs were all identified in the intron of annotated genes. Two SNPs, rs14920313 and rs14920355, were located in the doublecortin-like kinase 1 (*DCLK1*) gene, and rs313140585 was identified in the neurobeachin (*NBEA*) gene. In addition, two other genes, spastic paraplegia 20 (*SPG20*) and StAR-related lipid transfer domain containing 13 (*STARD13*), were found near the significant SNPs.

#### Ovary weight

Thirty-one SNPs showed significant association with ovary weight (Table 4), seven of which were located within a 700-kb region (169.01–169.71Mb) on GGA1. Furthermore, the other 24 SNPs were located in an 800-kb region spanning from 1.6 to 2.4 Mb on GGA28. Of the loci in GGA1, three were located in annotated genes, including NIMA-related kinase 5(*NEK5*), NIMA-related kinase 3(*NEK3*), and cytoskeleton associated protein 2 (*CKAP2*). Twelve of the 24 SNPs in GGA28 were located in known genes, and the remainders were located 0.4 to 10 kb away from the nearest known genes, which included 19 annotated genes (Table 4).

**Fig 2:**
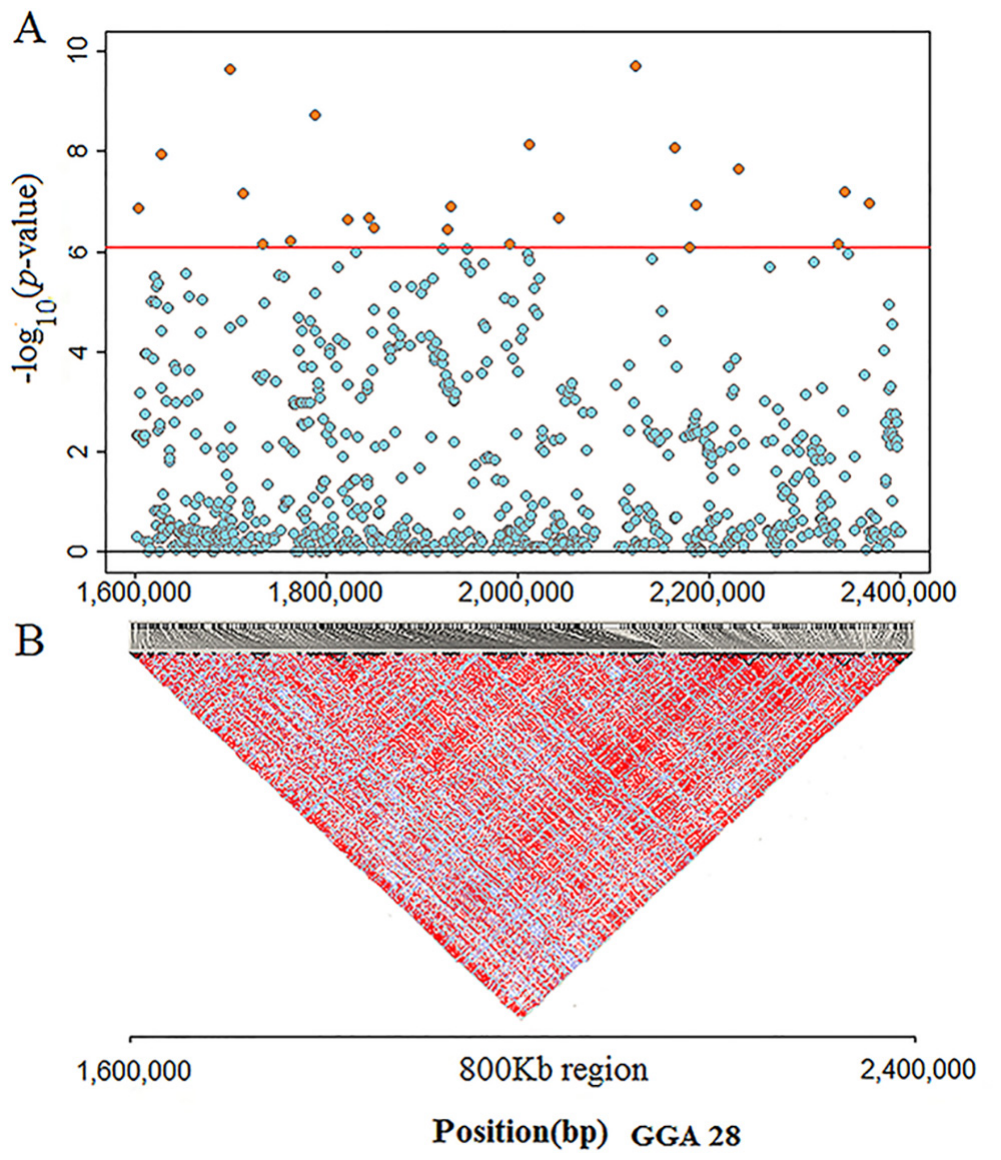
Regional plot for single nucleotide polymorphisms(SNPs) at GGA28 spanning from 1.60 to 2.40 Mb. Plot A: In the region of 1.60 to 2.40 Mb,744 SNPs (orange and blue points) were analyzed for their association with ovary weight (OW). The-log_10_ (observed *P* values) of the SNPs (y-axis) are presented according to their chromosomal positions (x-axis). Twenty-four SNPs (orange points) reached genome-wide significance level (black line, 8.43 × 10^−7^). **Plot B**: Seventy-six small-scale blocks were observed in this region.

Considering the large number of significant SNPs (24 loci) in a narrow region (800kb) in GGA28, we speculated that strong LD exists in this region. Hence, the LD analysis was performed in the genomic region from 1.60 to 2.40 Mb, which contained 744 SNPs, and 76 small scale blocks were observed in this region (S2 Table). Eight out of the 24 significant SNPs fell into six blocks, and the remaining 18 SNPs were not located in any of these blocks (Fig 2 and S2 Table). The results indicated that the significant loci in GGA28 were in poor LD with each other and that the loci may play independent roles.

## Discussion

The population used for this study was anF_2_ cross population, which could maximize the differences of the traits and increase the power to identify the QTLs for traits that differed between the breeds. Compared with previous association analyses in chickens, our analysis used a higher density (600K) SNP array covering chromosomes 1–28 and two unassigned linkage groups. In addition, this F_2_ population consisted of 1,512 hens, which is the largest population used for GWA analysis of egg traits so far, and therefore, the novel genomic region and loci revealed by the current study should be accurate and reliable. Several previous studies detected causal genomic regions or genes that controlled yolk weight using GWA analysis [17, 18]. However, these studies utilized phenotypes from limited age points, assuming that the genetic architecture underlying this trait does not vary with the aging process. In the current study, yolk weights of 11 age points from the onset of egg laying to 72 weeks of age were employed to detect the dynamic genetic control for yolk weights. In addition, this study was the first GWA analysis for ovary and follicle weight in chicken.

### Yolk weight

By performing GWA analysis, we identified 11 associations with yolk weight at six age points at genome-wide significance levels (*P*<8.33 × 10^−7^). These significant loci, which occurred on GGA 1, 3, 5, 17, and 28, had no overlap with previously reported QTLs. This might due to the specially designed F_2_crosses used in this study. In addition, the significant loci for each age point varied completely, which was notable because it indicated that the genetic control for yolk weight was age-dependent. However, the genetic correlations among yolk weights at each age points were relatively high (Table 2), suggesting certain consistent genetic variants existed to control yolk weight along with the aging process. This contradiction might be explained by the existence of suggestive significant common genomic regions or loci that associated with yolk weight at different age points (S1 Table). In contrast, the 11 genome-wide significant SNPs were considered more powerful and specific loci for yolk weight. These loci were located near or in 16 annotated genes.

One candidate gene with a biologically plausible function is zygote arrest 1(*ZAR1*), which is downstream (2,140bp) of rs313116098, an SNP significantly associated with YW48.*ZAR1* is considered to be an oocyte-specific maternal-effect gene [32] and is thought to function in the initiation of embryogenesis in many vertebrate species including humans, pigs, cattle, sheep, mice, rats, and frogs [33]. Michailidis et al. (2010) revealed that this gene was preferentially expressed in chicken oocytes, ovaries, testes, and embryos during embryonic development [34]. This gene is related to the development of follicular oocytes; however, its involvement in yolk weight is not clear.

Three genes, stonin 2 (*STON2*), galactosylceramidase(*GALC*), and potassium channel, voltage-gated modifier subfamily G, member 3 (*KCNG3*) have functions related to nerve regulation. In fact, nerve networks have close relationships with ovarian follicle development. Specifically, the maturity and ovulation of the yolky follicle is profusely innervated by both adrenergic and cholinergic fibers [35]. *STON2*encodes a protein that participates in synaptic vesicle recycling through interaction with synaptotagmin 1, which is required for neurotransmission [36]. *GALC* encodes galactosylceramidase, which is related to globoid cell leukodystrophy in humans [37]. *KCNG3*encodes for the voltage-gated potassium (Kv) channels, which regulate neurotransmitter release, insulin secretion, and neuronal excitability [38]. Neurons are mainly present within the thecal layers of the largest follicles. These neurons provide numerous neurochemicals (catecholamines, neurotrophins, vasoactive intestinal peptide) to the follicle [39]. Therefore, the differences among yolk weights of first eggs may be partly led by the variable maturity of the sequential follicles that are regulated by the nerve networks. The remaining screened genes have no direct relevance with the yolk deposition orovarian follicle development, and this maybe due to insufficient knowledge about these genes in chickens.

### Follicle weight

Follicles that connect to the ovary by pedicles are the sites for yolk formation. Hence, the mass of ovarian follicles represent the capacity of sequential yolk production of laying hens. However, the phenotypic or genetic correlation coefficients between follicle weight and yolk weight at each age point were all very low, even with the yolk weight of 72 weeks of age. This indicated that the genetic architecture might be disparate for single yolk weights and the mass of existing ovarian follicles at a certain age.

Three loci (rs14920313, rs14920355, and rs313140585) in GGA1 were significantly associated with follicle weight, and two candidate genes, *SPG20* and *STARD13*, harbored or were near these loci. *SPG20* encodes a protein containing an MIT (microtubule interacting and trafficking molecule) domain and can cause protein translocation to the plasma membrane when stimulated with epidermal growth factor (EGF) [40], whereas EGF, as one of the main intra-ovarian hormones produced by the germinal disc and granulosa cells, can stimulate growth and reduce atresia in follicles [41, 42]. *STARD13* encodes a protein possessing C-terminal STAR-related lipid transfer domain, which is a classical type of lipid transporter [43, 44]. In mammals,15proteins, *STARD1*-*STARD15*, possess a START domain, and cholesterol, 25-hydroxycholesterol, phosphatidylcholine, phosphatidylethanolamine, and ceramides are ligands for *STARD1/STARD3/STARD5*, *STARD5*, *STARD2/STARD10*, *STARD10*, and *STARD11*, respectively [43]. In the current study, the *STARD13* gene was identified with follicle weight, indicating its crucial role in lipid metabolism in the process of yolk deposition, an association that needs further investigation.

### Ovary weight

The SNPs that were significantly associated with the follicle-free ovary weight were distributed on two chromosomes, GGA1 and GGA28. The loci on GGA1 (169.01–169.70Mb) were upstream from and only 3 Mb away from the loci (172.06–172.45Mb), which were related to follicle weight, whereas the candidate genes in these two regions were completely different. Six genes containing these loci of interest or located near these loci in GGA1 were identified. However, only one gene hasa function plausible for ovarian development. This gene is *DHRS12*, a gene that encodes the enzyme of the short-chain dehydrogenases/reductases (SDR) family, which metabolizes many different compounds, including steroid hormones [45]. The ovarian steroid hormones include estrogen, testosterone, and progesterone; these are synthesized by follicular interstitial and granulosa cells, and estrogen can stimulate the liver for the synthesis of the yolky lipid and protein precursors [2]. Hence, *DHRS12*is closely related to ovarian development.

For the loci in GGA 28, the most significant SNP, rs316105069(*P* = 1.7 × s3^-10^), was located in two genes, including alkaline ceramidase 1 (*ACER1b*) andacyl-CoA synthetase bubblegum family member 2 (*ACSBG2*). These two genes are closely related to the biological process of yolk formation, such as lipoprotein synthesis, lipid droplet promotion, and lipid carrier. *ACER1*can catalyze the hydrolysis of very long chain ceramides to sphingosine, while sphingosine is absorbed and converted to palmitic acid and then acylated into chylomicron triglycerides (TGs), the materials needed for the synthesis of lipoproteins [46]. Another gene, *ACSBG2*,encodes proteins belonging to the acyl-CoA synthetase family, which can channel lipids into nascent lipid droplets in mammals [47]. Claire et al. (2013) demonstrated that the *ACSBG2* gene is significantly associated with abdominal fat deposition and exhibited functions related to lipid metabolism in chickens [48].

## Conclusions

In summary, the current study reports for the first time a GWA analysis on ovary and follicle weights in chickens. Our results revealed 12, 3, and 31 genome-based significant SNPs for yolk, follicle, and ovary weight, respectively, by GWA analysis with a 600K high-density SNP array. The GWA analysis for yolk weights at multiple age points suggested that the genetic control for yolk weight is age-dependent. A list of candidate genes such as *ZAR1*, *STARD13*, *ACER1b*, *ACSBG2*, and *DHRS12* were identified for their plausible function in yolk and follicle developments. Our findings establish a foundation for follow-up studies and create a better understanding of the molecular controls involved in the development of the ovary and its hierarchy of follicles.

## Supporting Information

S1 Fig Manhattan plot (left) and quantile-quantile plot (right) of the observed *P* values for the yolk weight at 32 weeks (YW32). (TIF)

S2 Fig Manhattan plot (left) and quantile-quantile plot (right) of the observed *P* values for the yolk weight at 32 weeks (YW36). (TIF)

S3 Fig Manhattan plot (left) and quantile-quantile plot (right) of the observed *P* values for the yolk weight at 32 weeks (YW40). (TIF)

S4 Fig Manhattan plot (left) and quantile-quantile plot (right) of the observed *P* values for the yolk weight at 32 weeks (YW44). (TIF)

S5 Fig Manhattan plot (left) and quantile-quantile plot (right) of the observed *P* values for the yolk weight at 32 weeks (YW48). (TIF)

S6 Fig Manhattan plot (left) and quantile-quantile plot (right) of the observed *P* values for the yolk weight at 32 weeks (YW52). (TIF)

S7 Fig Manhattan plot (left) and quantile-quantile plot (right) of the observed *P* values for the yolk weight at 32 weeks (YW56). (TIF)

S8 Fig Manhattan plot (left) and quantile-quantile plot (right) of the observed *P* values for the yolk weight at 32 weeks (YW60). (TIF)

S9 Fig Manhattan plot (left) and quantile-quantile plot (right) of the observed *P* values for the yolk weight at 32 weeks (YW66). (TIF)

S10 Fig Manhattan plot (left) and quantile-quantile plot (right) of the observed *P* values for the yolk weight at 32 weeks (YW72). (TIF)

S11 Fig Manhattan plot (left) and quantile-quantile plot (right) of the observed *P* values for follicle weight (FW). (TIF)

S12 Fig Manhattan plot (left) and quantile-quantile plot (right) of the observed *P* values for ovary weight (OW). (TIF)

S1 Table Suggestive significant single nucleotide polymorphisms (SNPs) for yolk weight at different age points by univariate model. (XLSX)

S2 Table Detailed messages of 76 small scale blocks clustered from 744 SNPs from 1.60 to 2.40 Mb in GGA28. (XLSX)
