# Supplementary figures and images for: Promising Loci and Genes for Yolk and Ovary Weight in Chickens Revealed by a Genome-Wide Association Study
Source: PLoS One. 2015 Sep 2;10(9):e0137145. doi: 10.1371/journal.pone.0137145 (PMC4558091; doi:10.1371/journal.pone.0137145)

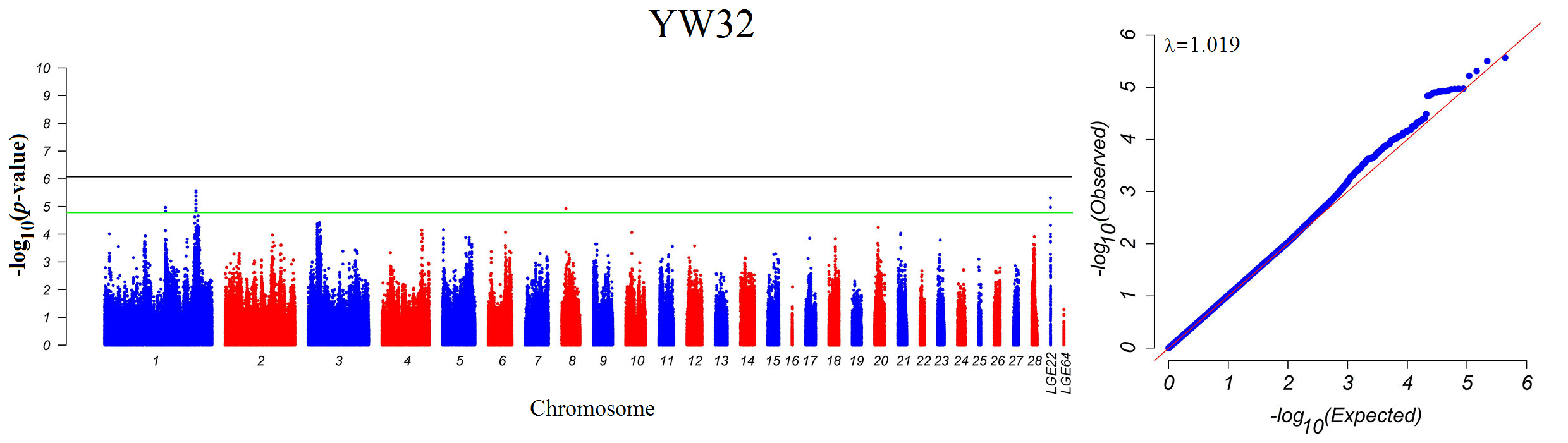

Supplement: S1 Fig — (TIF) [file pone.0137145.s001.tif]

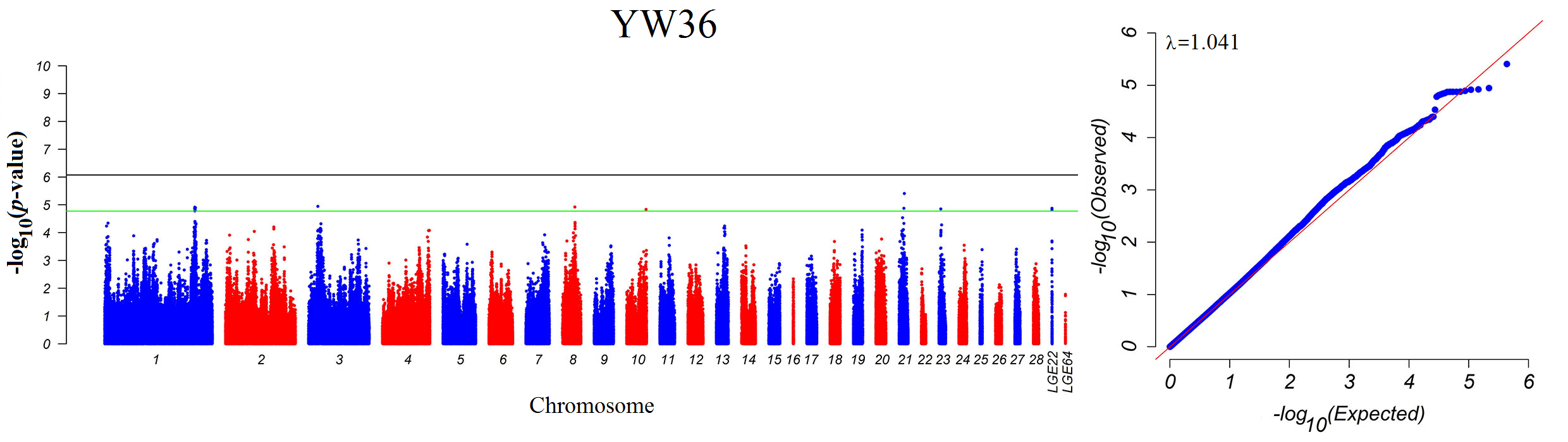

Supplement: S2 Fig — (TIF) [file pone.0137145.s002.tif]

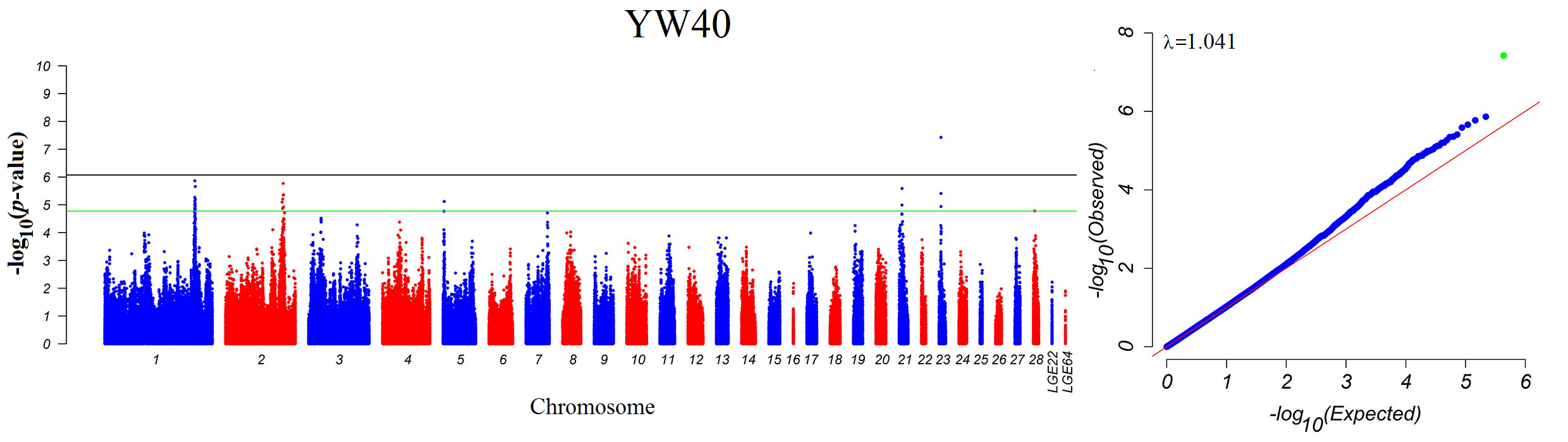

Supplement: S3 Fig — (TIF) [file pone.0137145.s003.tif]

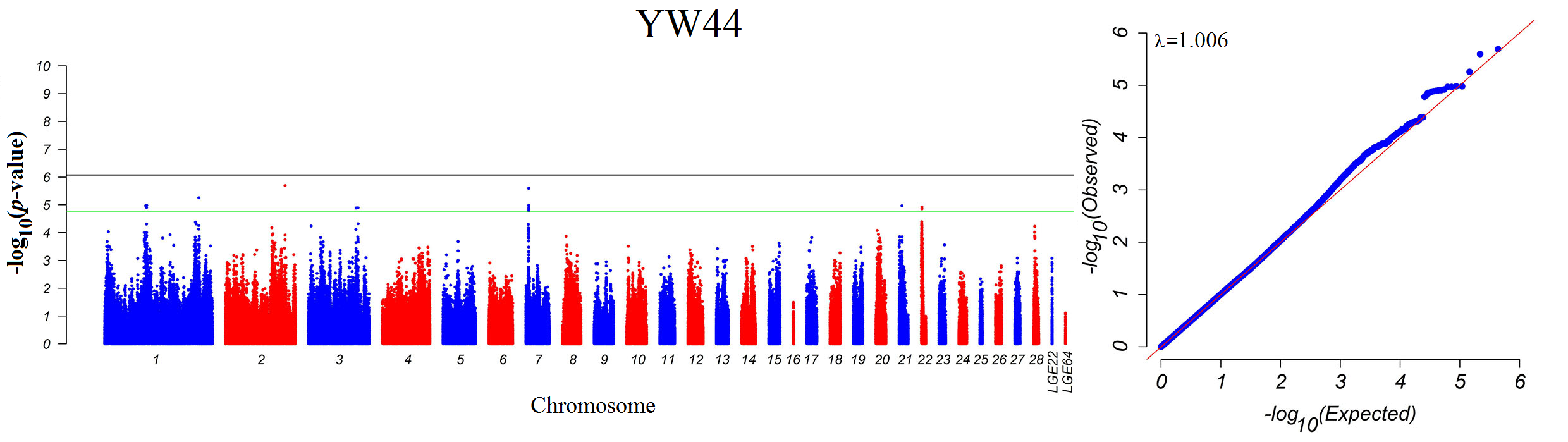

Supplement: S4 Fig — (TIF) [file pone.0137145.s004.tif]

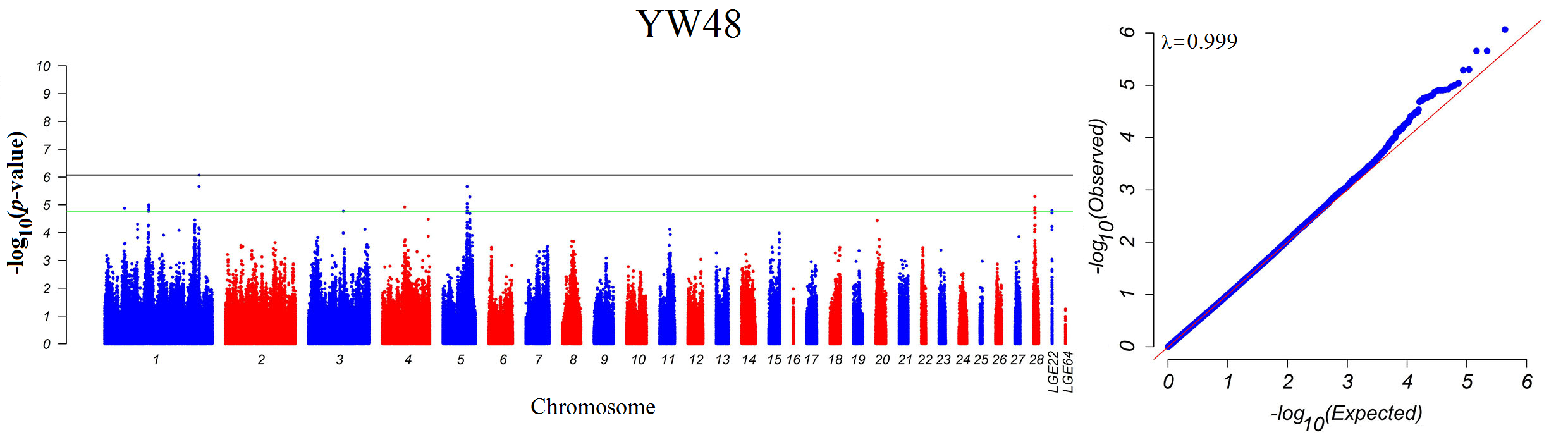

Supplement: S5 Fig — (TIF) [file pone.0137145.s005.tif]

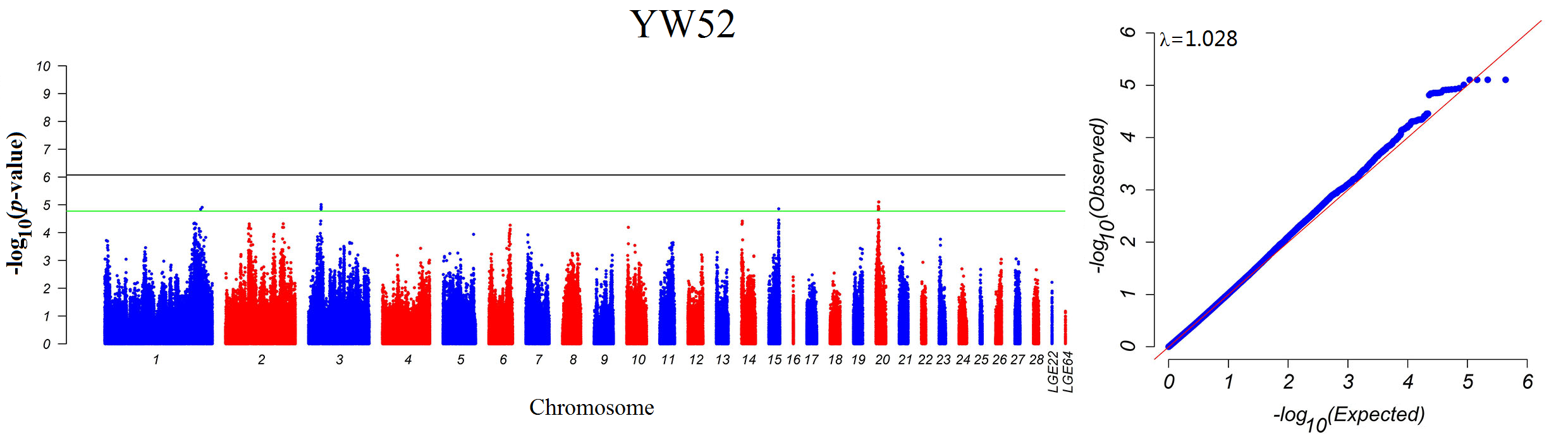

Supplement: S6 Fig — (TIF) [file pone.0137145.s006.tif]

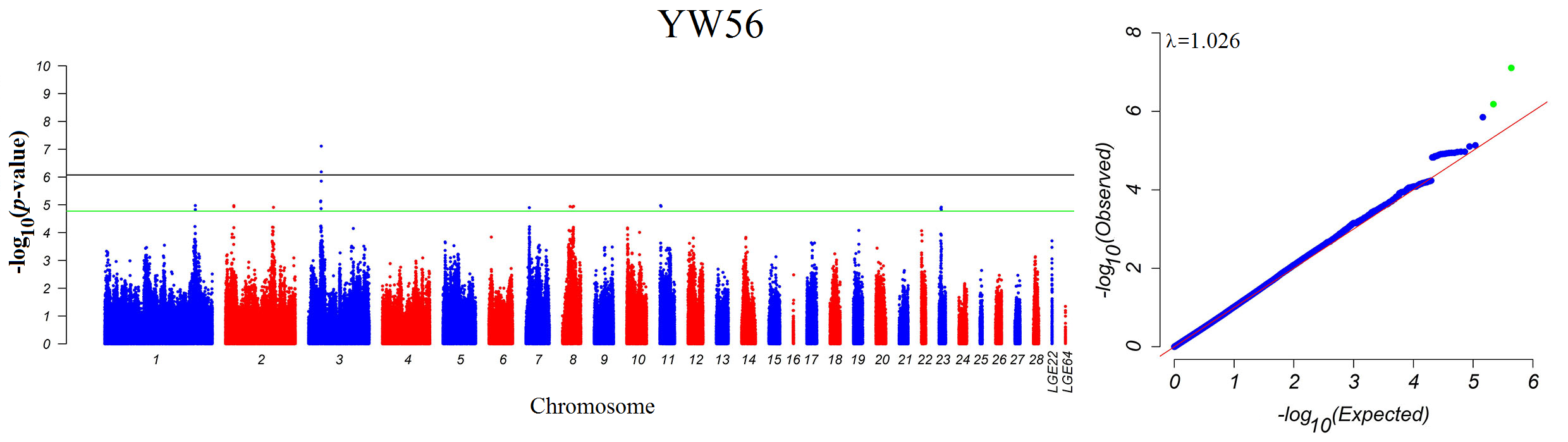

Supplement: S7 Fig — (TIF) [file pone.0137145.s007.tif]

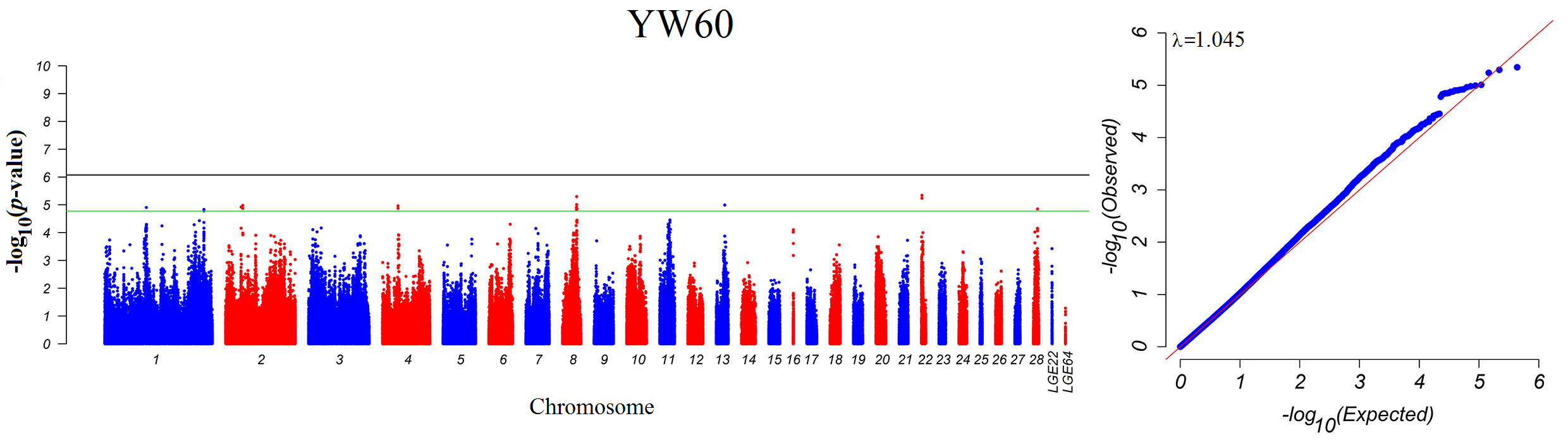

Supplement: S8 Fig — (TIF) [file pone.0137145.s008.tif]

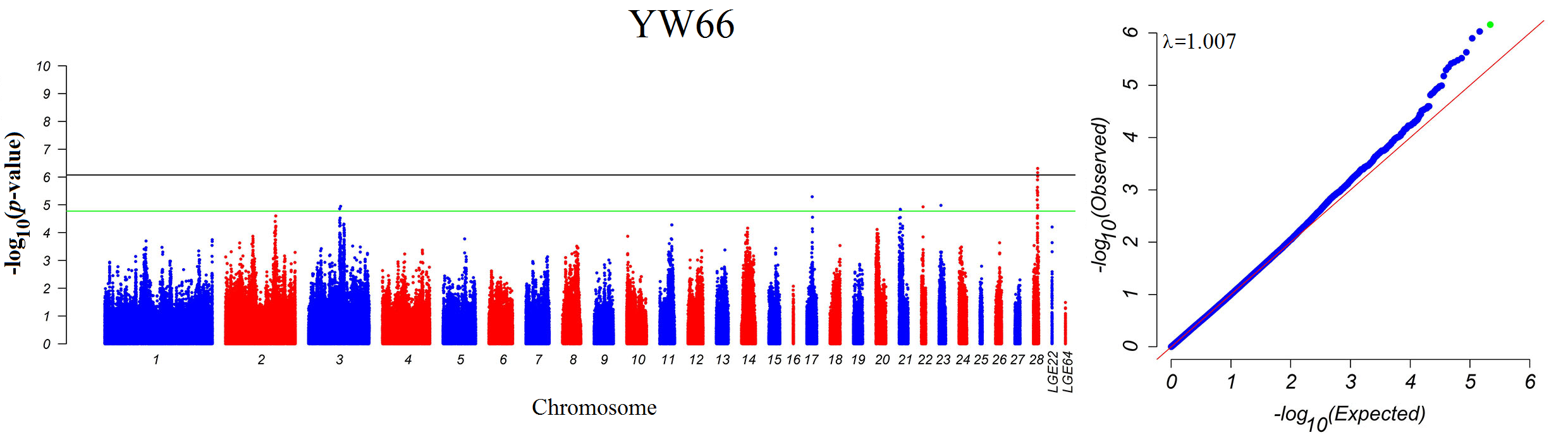

Supplement: S9 Fig — (TIF) [file pone.0137145.s009.tif]

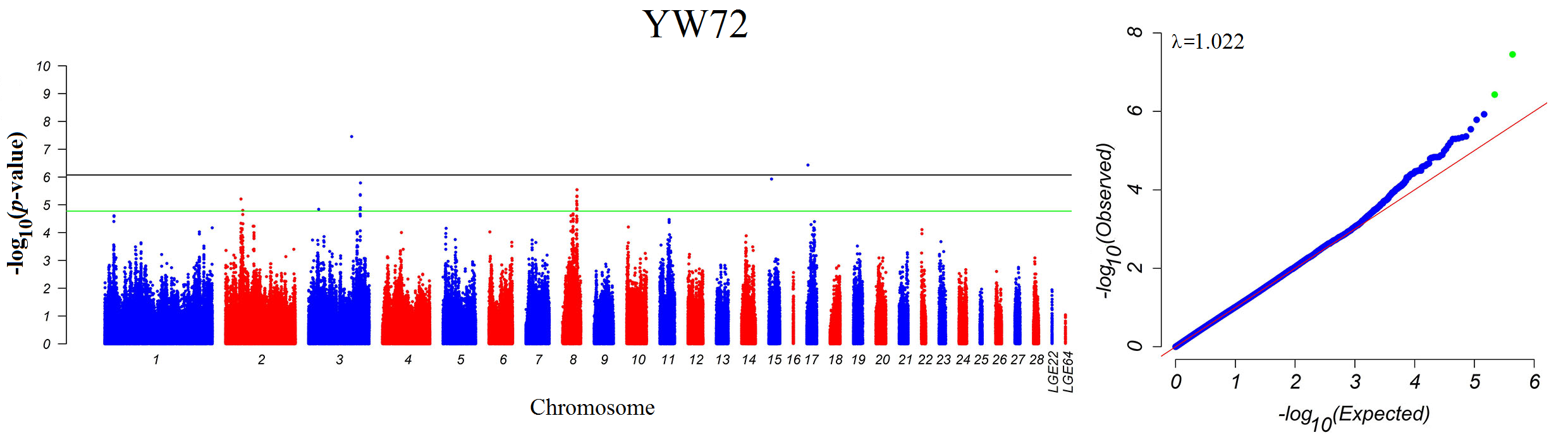

Supplement: S10 Fig — (TIF) [file pone.0137145.s010.tif]

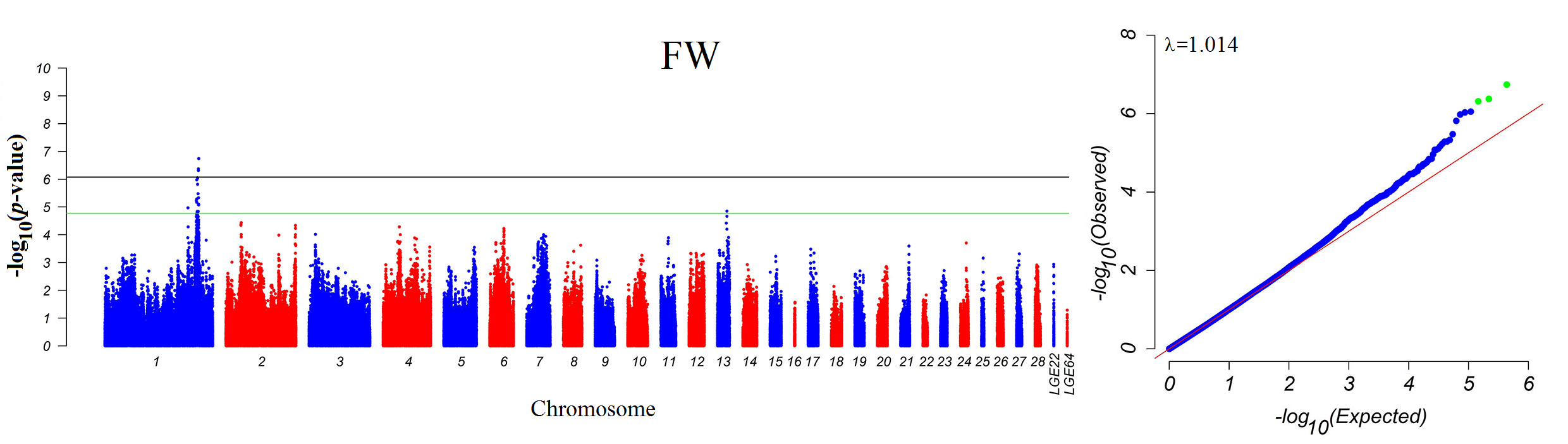

Supplement: S11 Fig — (TIF) [file pone.0137145.s011.tif]

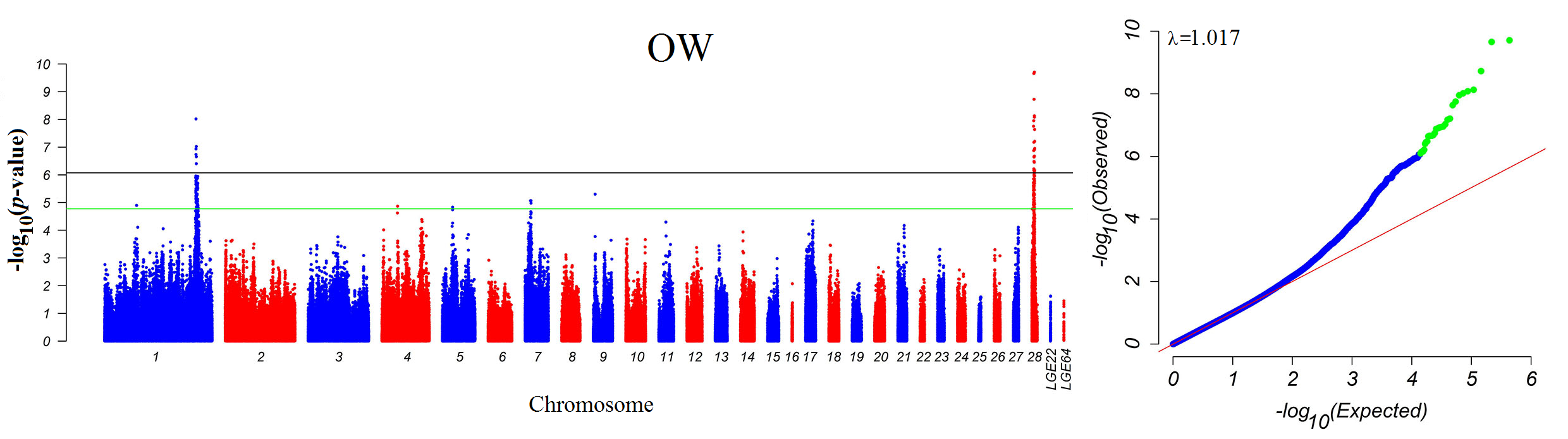

Supplement: S12 Fig — (TIF) [file pone.0137145.s012.tif]
